# Application and efficacy of a self-made minimally invasive transverse cross-link in percutaneous pedicle screw surgery

**DOI:** 10.1097/MD.0000000000044046

**Published:** 2025-08-29

**Authors:** Yue Wang, Chenzhao Liu, Xiaopeng Zheng, Shijie Chen, Honghan Huang, Guangfeng Ling, Jianquan Chen, Chun Wang, Liangsheng Li

**Affiliations:** a Department of Spinal Surgery, Mindong Hospital Affiliated to Fujian Medical University, Fujian, China.

**Keywords:** minimally invasive spine surgery, minimally invasive transverse cross-link, percutaneous pedicle screw fixation, thoracolumbar fracture

## Abstract

To design a minimally invasive transverse cross-link for use in percutaneous pedicle screw fixation surgery and to explore and observe its clinical application value. A retrospective analysis of the clinical data of 50 patients with thoracolumbar burst fractures treated with percutaneous pedicle screws was conducted. A self-made, minimally invasive transverse cross-link was implanted during surgery. Preoperative, postoperative, and final follow-up imaging examinations were performed to compare the imaging parameters and document the occurrence of complications related to internal fixation. Clinical efficacy was evaluated via the visual analog scale for pain and the Oswestry disability index. All patients successfully underwent surgery and achieved bony fusion 1 year after surgery. Compared with the preoperative values, the local Cobb angle and anterior vertebral height ratio at 3 days, 3 months, and final follow-up significantly improved (*P* < .05). The visual analog scale and Oswestry disability index scores at 3 months and at the final follow-up were significantly better than the preoperative scores (*P* < .05). No complications related to internal fixation, including screw loosening, withdrawal, or rod fracture, were observed during the follow-up period. The application of the self-made minimally invasive transverse cross-link provides additional stability to percutaneous pedicle screw fixation surgery while maintaining the integrity of the posterior midline structures. This technique is safe and feasible.

## 1. Introduction

With the gradual development of minimally invasive spinal techniques, the application of percutaneous pedicle screw fixation has become increasingly widespread in various types of spinal surgery.^[1,2]^ This technique is characterized by minimal trauma, reduced blood loss, shorter surgery times, faster postoperative recovery, and lower infection rates.^[3–5]^ The internal fixation devices used in spinal surgery include pedicle screws connected vertically by rods, as well as transverse cross-links that secure 2 rods together.^[6]^

In traditional open surgeries, transverse cross-links can be easily installed through exposed and disrupted midline structures. However, in percutaneous pedicle screw surgery, the small incision and preserved posterior spinal structures make the use of conventional transverse cross-links impossible.^[7]^

**Figure 1.**
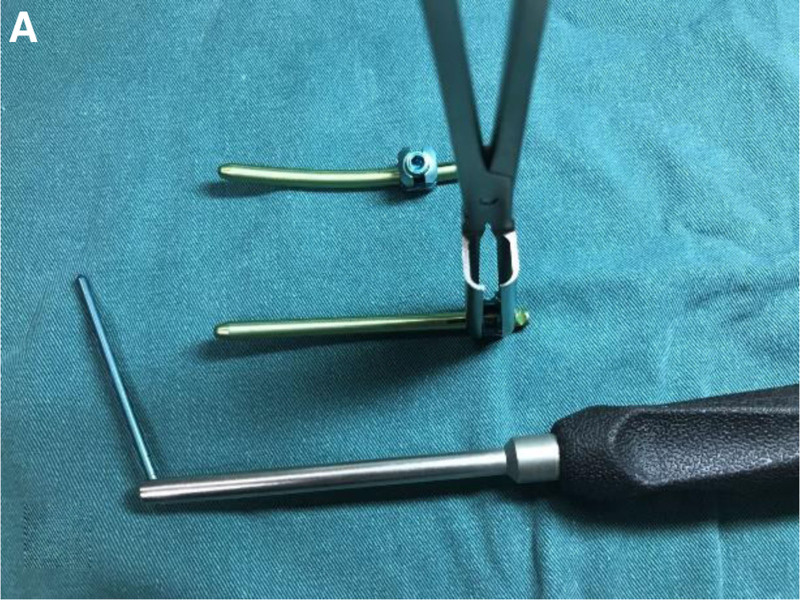
Components of the novel minimally invasive transverse cross-link: a pair of connecting blocks on both sides of the spine, a spinous process drill, a transverse cross-link, a rod holder, nuts, etc.

To address this challenge, our research team independently designed a novel minimally invasive transverse cross-link (patent numbers: CN201710455633.5; CN202120975176.4), which can be installed through the original surgical incision used for screw placement without disrupting the integrity of the midline structures (Fig. 1). This study retrospectively analyzed 50 cases of thoracolumbar burst fractures treated with a self-made minimally invasive transverse cross-link combined with percutaneous pedicle screw fixation from January 2018 to June 2022. The outcomes were satisfactory, as reported below.

## 2. Materials and methods

### 2.1. Inclusion and exclusion criteria

Inclusion criteria:

1. Age between 18 and 60 years;
2. Single-segment thoracolumbar vertebral fractures involving T11–L2, with no neurological deficits;
3. Fractures caused by clear traumatic factors;
4. Fresh traumatic fractures;
5. TLICS score ≥4 points;
6. Follow-up of at least 1 year with complete clinical and radiological data;
7. Patients signed an informed consent form.

Exclusion criteria:

1. Pathological fractures (including tuberculosis, primary or metastatic tumors, etc);
2. Spinal canal occupation or associated neurological injury requiring open spinal decompression surgery;
3. Severe underlying conditions that preclude surgery.

A total of 50 patients, consisting of 23 males and 27 females, with an average age of 44.84 ± 8.20 years, were included in the study. The average time from injury to surgery was 3.24 ± 0.94 days.

Injury locations:

- T11 vertebra: 4 patients.
- T12 vertebra: 15 patients.
- L1 vertebra: 18 patients.
- L2 vertebra: 13 patients.

Causes of injury:

- Falls from height: 19 patients.
- Traffic accidents: 15 patients.
- Other injuries: 16 patients.

All patients had no neurological deficits and underwent percutaneous pedicle screw fixation surgery with 6 screws (intra-segmental).

This study was approved by the Ethics Committee of the Affiliated Ningde Hospital of Fujian Medical University (Ethical approval no. 2023041101K), and all patients provided written informed consent. The study was conducted in accordance with the principles of the Declaration of Helsinki.

### 2.2. Surgical procedure

After general anesthesia, the patient is placed in the prone position with the abdomen suspended and the spine in a posteriorly extended position. Under fluoroscopic guidance, the positions of the pedicles to be instrumented are marked. A 1.5 to 2.0 cm incision is made, followed by dissection of the skin and fascial layers, with blunt dissection of muscle fibers using the fingers. Classic percutaneous pedicle screw insertion techniques are employed, with pedicle screws inserted sequentially. A connecting rod is appropriately contoured to match the required spinal curvature, and the rod is installed via an external extension arm. After proper distraction and reduction are achieved, the screw caps are tightened.

**Figure 2.**
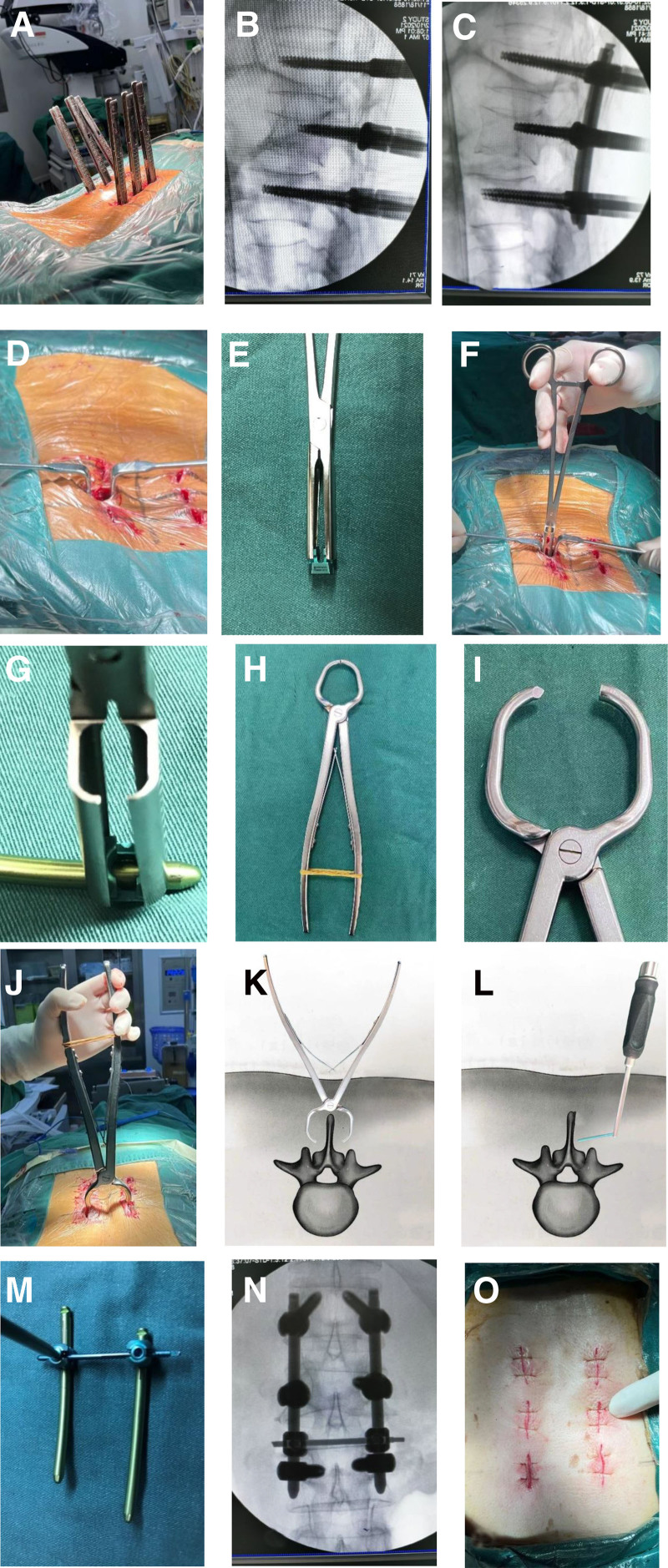
The detailed steps of surgery. (A–C) Standard insertion of percutaneous pedicle screws; (D) removal or fracture of the screw extension sleeve, exposing the area below the injured vertebral screws; (E–G) sequential placement of transverse connecting clamps; (H–K) use of a spinous process drill to create a pathway at the appropriate level in the interspinous ligament or spinous process; (L) insertion of the connecting rod through the established pathway into the bilateral clamps via a percutaneous rod holder; (M, N) Tightening of the bilateral clamp caps; and (O) surgical incision.

Next, the screw extension sleeve is removed or fractured. At the shallowest point beneath the skin, a pathway is created via a spinous process drill at a level above or below the pedicle screws, which target the interspinous ligament or spinous process. A retractor is used to expose the bilateral connecting rods at this location, and transverse connecting clamps are then installed. Finally, a percutaneous rod holder is used to insert the appropriately sized connecting rod through the established transverse pathway into the bilateral clamps, and the clamp caps are sequentially tightened. The detailed steps are shown in Figure 2.

### 2.3. Postoperative management and follow-up schedule

Vital signs are closely monitored, pain relief and anti-infection treatments are provided, and regular repositioning and ankle pump exercises are performed to prevent pressure ulcers and deep vein thrombosis in the lower limbs. Two days after surgery, the patient was assisted in walking via a waist brace and guided in lumbar and dorsal muscle function exercises. Additionally, education on rehabilitation-related knowledge should be provided. Follow-ups were conducted for at least 12 months.

### 2.4. Radiologic evaluation and clinical assessment

#### 2.4.1. Radiologic parameters

Radiologic parameters included the measurement of the local Cobb angle (LKCA) on x-ray images, defined as the angle between the superior endplate of the upper vertebra and the inferior endplate of the lower vertebra, and the anterior vertebral height ratio (AVHR), calculated as the ratio of the actual anterior vertebral height of the injured vertebra to the average height of the adjacent superior and inferior vertebra. All patients underwent x-ray imaging of the thoracolumbar spine in the anteroposterior view preoperatively, at 3 days postoperatively, 3 months postoperatively, and at the final follow-up, and any complications related to internal fixation (including screw loosening, rod breakage, and transverse cross-link fractures) were observed.

#### 2.4.2. Clinical parameters

The clinical evaluation parameters included the visual analog scale (VAS) for pain and the Oswestry disability index (ODI) for functional recovery. VAS scores were used to assess lumbar pain, whereas ODI scores were used to evaluate functional recovery. VAS and ODI scores were calculated preoperatively, 3 days postoperatively, 3 months postoperatively, and at the final follow-up.

### 2.5. Statistical analysis

Statistical analysis was performed via SPSS 25.0 software (IBM Corporation, Chicago). For normally distributed continuous data, the mean and standard deviation were used for description, and paired *t*-tests were applied for comparisons. For non-normally distributed continuous data, the median and interquartile range were used for description, and the Wilcoxon signed-rank test was employed for comparisons. To account for the time-progressive repeatability comparison, the *P*-values for multiple tests were corrected using the Bonferroni method to control for Type I errors. Categorical data are expressed as frequencies and percentages. A *P* value of <.05 was considered statistically significant.

Missing data for the study were minimal (<5%) and were handled using multiple imputation to ensure completeness. The follow-up information was available for all 50 patients, with 100% follow-up completion at the final assessment, further supporting the reliability of the data.

## 3. Results

### 3.1. Demographic data

**Figure 3.**
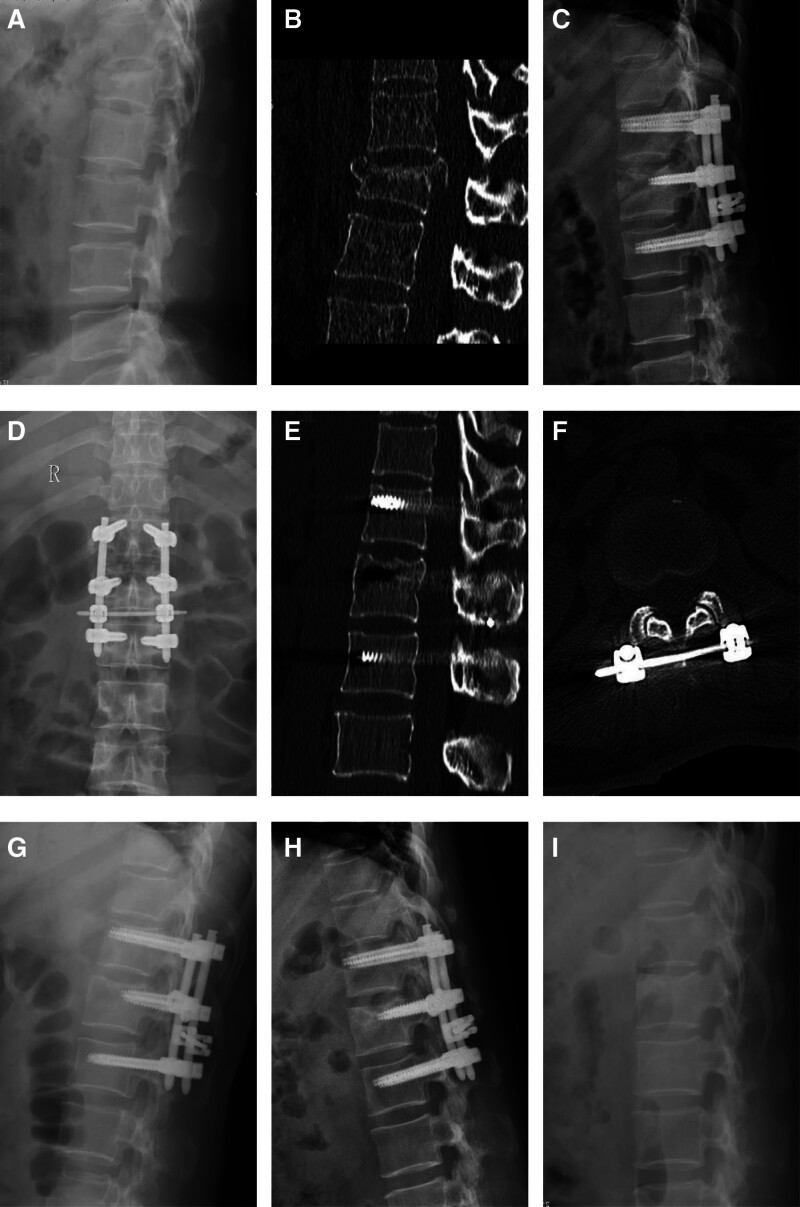
Imaging data of a typical patient in the CL group. (A) Preoperative lateral x-ray image showing that the L1 vertebra was wedge shaped; (B) preoperative axial CT image showing that the fracture line involved the middle column and that the bone fragment protruded into the spinal canal; (C, D) postoperative x-ray anteroposterior and lateral views showing that the position of internal fixation was excellent (1 wk after the operation); (E, F) postoperative CT image showing that the channel of the connecting rod of the minimally invasive transverse cross-link was located below the spinous process of the L1 vertebra (1 mo after the operation); (G) postoperative follow-up image showing that the height of the injured vertebra was well maintained (1 yr after the operation); (H) the 1-year follow-up image showing that the height of the injured vertebra was well maintained and that no significant loss of correction was found; and (I) x-ray images after removal of internal fixation. CL = cross-link, CT = computed tomography.

A total of 50 patients included in the study successfully underwent surgery and were followed up for more than 12 months, with an average follow-up of 17.2 ± 4.6 months and a minimum follow-up of 12 months. After bony fusion was confirmed, all patients returned to the hospital for removal of the internal fixation devices. No complications, such as screw loosening, rod breakage, or transverse cross-link fracture, were observed. A typical case is shown in Figure 3.

### 3.2. Radiologic and clinical outcomes

Compared with the preoperative values, both groups demonstrated a significant decrease in the LKCA and a significant increase in the AVHR at 3 days postoperatively, 3 months postoperatively, and at the final follow-up (*P* < .05). However, compared with the immediate postoperative values, both the LKCA and AVHR showed partial loss of correction at the final follow-up, with statistically significant differences (*P* < .05; see Table 1). This phenomenon may be related to the postoperative stress redistribution and the “shell phenomenon” during bone healing, but it has no significant impact on the final function. Compared with the preoperative scores, the VAS and ODI scores at 3 months postoperatively and at the final follow-up were significantly improved. No significant differences were observed in the VAS or ODI scores between the 3-month and final follow-up assessments (*P* > .05; see Table 2).

**Table 1:** Comparison of radiologic data (*x̄* ± *s*).

Time	LKCA (°)	AVHR (%)
Preoperative	18.5 ± 5.6	57.2 ± 8.0
3 d postoperative	−0.7 ± 6.0*	100 ± 3.3*
3 mo postoperative	−0.2 ± 5.7*^,^**	100 ± 2.1*^,^**
Final follow-up	−0.1 ± 10.1*^,^**^,^***	99.1 ± 2.6*^,^**^,^***
*F*	90.49	1237.17
*P*	<.001	<.001

Compared with the preoperative values, *P < .01.

Compared with 3 d after the operation, **P < .01.

Compared with 3 mo after operation, ***P < .01.

LKCA = local Cobb angle, AVHR = anterior vertebral height ratio.

**Table 2:** Comparison of follow-up data (*x̄* ± *s*).

Time	VAS	ODI (%)
Preoperative	7.72 ± 0.70	82.74 ± 4.49
3 mo postoperative	1.38 ± 0.60*	20.32 ± 2.33*
Final follow-up	0.80 ± 0.57*^,^**	16.08 ± 2.55*^,^**
*F*	1871.98	6495.64
*P*	<.001	<.001

Compared with the preoperative values, *P < .01.

Compared with 3 mo after the operation, **P < .01.

ODI = Oswestry disability index, VAS = visual analog scale.

Patient, female, 47 years old, L1 burst fracture, TLICS score: 4, treated with transverse cross-linking combined with short-segment percutaneous pedicle screw fixation (Fig. 3).

## 4. Discussion

Pedicle screw fixation technology enables fixation of the anterior, middle, and posterior columns of the vertebra, thereby restoring the normal alignment of the spine, reconstructing, and maintaining spinal stability immediately. It is widely applied in the treatment of various spinal diseases, including degenerative conditions, tumors, and trauma, with well-documented efficacy.^[8]^

Traditional open pedicle screw fixation requires extensive dissection of paraspinal muscle tissue and posterior spinal structures to place the pedicle screws. This results in significant trauma, substantial blood loss, and potential postoperative complications such as ischemic necrosis and muscle fibrosis, which can lead to chronic low back pain.

In contrast, percutaneous pedicle screw fixation offers notable advantages in these aspects.^[9]^ Not only does it preserve the benefits of open surgery – such as satisfactory reduction and strong fixation – but it also significantly reduces damage to posterior lumbar structures, particularly the posterior ligamentous complex (PLC). The PLC, consisting of the facet joint capsules, ligamentum flavum, interspinous ligaments, and supraspinous ligaments, is crucial for limiting excessive flexion, rotation, displacement, and separation of the spine, thus playing an essential role in spinal stability.^[10]^ In open spinal surgeries or decompression procedures, the PLC is often disrupted or completely excised at specific levels. The primary advantage of percutaneous surgery is the preservation of this reliable stabilizing structure. Therefore, compared with open surgery, percutaneous procedures result in less trauma, less blood loss, shorter surgery times, and faster recovery and are widely recognized.

Traditionally, pedicle screw systems include vertically connected rods, with transverse cross-links locking the 2 vertical rods across the midline. Biomechanical studies have shown that the use of cross-links can connect the separately placed rods into an “H”-shaped parallelogram structure, improving axial rotational stability. This enhances the system’s ability to withstand higher loads and reduces the incidence of screw loosening.^[11,12]^ Additionally, the use of a second transverse cross-link provides an additive effect on axial stability, making the use of 2 cross-links preferable to 1 in long-segment fixation.^[13]^ In traditional open posterior surgeries, transverse cross-links can be easily installed through exposed and disrupted midline structures. However, in percutaneous pedicle screw surgery, the small incision and preserved posterior spinal structures make it difficult to place conventional transverse cross-links.

Kyle et al^[7]^ conducted a cadaveric feasibility study in which transverse cross-links were successfully installed in percutaneous pedicle screw surgery without disrupting the PLC or removing the spinous processes. However, this technique has only been tested on cadavers and has not been clinically validated. Furthermore, a new posterior lateral incision must be created via fluoroscopic guidance, and the incision must be drilled transversely through the muscle and bone to place and secure the transverse cross-link. This method is technically challenging, involves greater surgical trauma, requires more fluoroscopy, and theoretically carries a greater surgical risk.

To address these challenges, our research team developed a novel minimally invasive transverse cross-link that can be placed through the original surgical incision used for percutaneous pedicle screw fixation without the need for additional incisions. This approach is suitable for both open and minimally invasive spinal surgeries where the integrity of the midline structure is preserved. Minimally invasive transverse cross-linking has been biomechanically validated,^[14]^ patented, and successfully applied in various types of pedicle screw fixation surgeries, consistently achieving satisfactory clinical outcomes. The slight decline in imaging parameters, such as the LKCA and AVHR, observed at the final follow-up compared with the immediate postoperative values, may be related to postoperative stress redistribution and the “shell phenomenon” during bone healing. However, this change did not have a significant impact on the final functional outcomes, as evidenced by the continued improvement in VAS and ODI scores.

The thoracolumbar vertebra (T11–L2) are located at the junction of the physiological movement of the thoracolumbar spine, making them a site of concentrated stress, which predisposes them to fractures, accounting for approximately 60% to 70% of all spinal fractures.^[15]^ Additionally, thoracolumbar burst fractures with PLC injury (TLICS ≥ 4) result in severe 3-column damage and spinal instability, necessitating high demands for the stability of pedicle screw fixation. Therefore, this study focused on patients with this specific type of thoracolumbar fracture, which more accurately reflects the value of transverse cross-linking. The results of this study demonstrated that all patients showed significant improvements in the local Cobb angle (LKCA) and AVHR postoperatively compared with the preoperative values. Furthermore, both the VAS and ODI scores improved significantly. At the 1-year follow-up, all patients had achieved bony fusion, and no complications related to internal fixation occurred, effectively confirming the reliable efficacy of percutaneous pedicle screw fixation in treating thoracolumbar fractures. Moreover, at the 1-year follow-up, both the LKCA and AVHR showed a slight loss of correction compared with the immediate postoperative values. This loss may be related to the “shell phenomenon”^[16,17]^; however, it does not affect the clinical outcomes of thoracolumbar fracture patients.^[18]^

One of the limitations of this study is the lack of a control group, which restricts our ability to directly compare the outcomes of our novel minimally invasive transverse cross-link with other treatment options. The absence of a control group was due to ethical considerations and the practical challenges of implementing a randomized controlled trial in this setting. Ethical concerns related to withholding a potentially beneficial intervention from patients led to the decision to perform a single-group retrospective analysis. However, we acknowledge that a prospective controlled study would provide stronger evidence of the efficacy and safety of this technique. Future research plans include conducting prospective, randomized controlled trials to further validate our findings and compare the clinical outcomes of our novel approach with conventional methods.

## 5. Conclusion

This study presents a novel minimally invasive transverse cross-link technique, which enhances the stability of percutaneous pedicle screw fixation without requiring additional incisions, while preserving the integrity of the midline structures. This approach is particularly suitable for minimally invasive surgeries that aim to retain the PLC, offering a cost-effective and efficient alternative to traditional methods. By reducing surgical trauma and preserving key spinal structures, it holds potential for improving clinical outcomes in spinal fracture treatments. With further prospective studies, this technique may become a valuable addition to clinical practice, especially in cases requiring stable fixation with minimal disruption to surrounding tissues.

## Author contributions

**Conceptualization:** Yue Wang, Chenzhao Liu, Chun Wang, Liangsheng Li.

**Data curation:** Yue Wang, Chenzhao Liu, Xiaopeng Zheng, Guangfeng Ling, Jianquan Chen, Chun Wang.

**Formal analysis:** Yue Wang, Chenzhao Liu, Xiaopeng Zheng, Jianquan Chen, Chun Wang, Liangsheng Li.

**Funding acquisition:** Liangsheng Li.

**Investigation:** Yue Wang, Chenzhao Liu, Xiaopeng Zheng, Shijie Chen, Honghan Huang, Guangfeng Ling, Jianquan Chen, Chun Wang, Liangsheng Li.

**Methodology:** Yue Wang, Chenzhao Liu, Xiaopeng Zheng, Shijie Chen, Guangfeng Ling, Chun Wang, Liangsheng Li.

**Resources:** Shijie Chen, Honghan Huang.

**Software:** Shijie Chen, Jianquan Chen.

**Supervision:** Yue Wang, Xiaopeng Zheng, Honghan Huang, Guangfeng Ling, Jianquan Chen.

**Validation:** Yue Wang, Honghan Huang, Guangfeng Ling, Chun Wang, Liangsheng Li.

**Visualization:** Honghan Huang, Chun Wang, Liangsheng Li.

**Writing** – **original draft:** Yue Wang, Chun Wang, Liangsheng Li.

**Writing** – **review & editing:** Yue Wang, Chun Wang, Liangsheng Li.
